# Sneaky Diagnosis of Pleural Malignant Mesothelioma in Thoracic Surgery: All That Glitters Is Not Gold

**DOI:** 10.3390/jcm11113225

**Published:** 2022-06-06

**Authors:** Riccardo Orlandi, Francesca Bono, Diego Luigi Cortinovis, Giuseppe Cardillo, Ugo Cioffi, Angelo Guttadauro, Emanuele Pirondini, Stefania Canova, Enrico Mario Cassina, Federico Raveglia

**Affiliations:** 1 Department of Thoracic Surgery, ASST Monza, San Gerardo Hospital, 20900 Monza, Italy; e.pirondini@asst-monza.it (E.P.); e.cassina@asst-monza.it (E.M.C.); federico.raveglia@asst-monza.it (F.R.); 2 Department of Pathology, ASST Monza, University of Milan-Bicocca, 20900 Monza, Italy; polletta.79@libero.it; 3 Department of Medical Oncology, ASST Monza, University of Milan-Bicocca, 20900 Monza, Italy; d.cortinovis@asst-monza.it (D.L.C.); s.canova@asst-monza.it (S.C.); 4 Department of Thoracic Surgery, AO San Camillo-Forlanini, 00152 Rome, Italy; gcardillo@scamilloforlanini.rm.it; 5 Department of Surgery, University of Milan, 20122 Milan, Italy; ugo.cioffi@guest.unimi.it; 6 Department of Medicine and Surgery, Istituti Clinici Zucchi, University of Milan-Bicocca, 20900 Monza, Italy; angelo.guttadauro@unimib.it

**Keywords:** mesothelioma, pleura, BAP1, hydropneumothorax, in situ, immunohistochemistry

## Abstract

Malignant Pleural Mesothelioma (MPM) is a highly aggressive disease whose diagnosis could be challenging and confusing. It could occur with atypical presentations on every examined level. Here, we present three unconventional cases of the complex diagnostic process of MPM that we have experienced during routine practice: a patient with reactive mesothelial hyperplasia mimicking MPM, an unexpected presentation of MPM with persistent unilateral hydropneumothorax, a rare case of MPM in situ. Then, we review the relevant literature on each of these topics. Definitive biomarkers to confidently distinguish MPM from other pleural affections are still demanded. Patients presenting with persistent hydropneumothorax must always be investigated for MPM. MPM in situ is now a reality, and this raises questions about its management.

## 1. Introduction

Malignant Pleural Mesothelioma (MPM) is an infrequent intrathoracic tumor, accounting for less than 1% of all oncological diseases, though representing the most frequent primary malignant neoplasia affecting the pleura and the second pleural malignancy overall, after pleural metastasis from epithelial cancers [1]. MPM rises from mesothelial cells of the pleura, which originate from mesoderm, representing the primitive celoma coating. It has a recognized pathogenetic factor: about 80% of patients with MPM have been exposed to asbestos fiber, with a latency time of at least 20–40 years from occupational or occasional exposure to the onset of disease [2]. MPM has a worldwide incidence of 10–30 cases per million, and in Italy, 1500–2000 new cases per year are diagnosed [3]. In developed countries, the peak incidence is expected around the 2020s [4] due to the wide use of asbestos after the Second World War and its following ban since the 1990s in western countries. However, this is not the case in developing countries, where asbestos is still commonly used, and the peak may follow several years from now there. The disease is more frequent in males, mainly because of the more common occupational exposure, with a median age of 70 years [5]. Patients usually report mild and non-specific symptoms such as dyspnea and cough due to pleural effusion and/or dull chest pain. Definitive diagnosis usually requires histological specimens through biopsy [2]. It can be suspected directly on morphology through hematoxylin-eosin (H&E); however, pathologists generally recommend confirmation through immunohistochemistry (IHC). Calretinin, Wilms tumor 1 (WT-1), cytokeratin 5 (CK5), podoplanin, mesothelin, and heart development protein with EGF like domains 1 (HEG1) are immunohistochemical biomarkers of mesothelial differentiation, whereas carcinoembryonic antigen (CEA), B72.3, Ber-EP4, Lewis^y^ blood group (BG8), MOC-31, CD15, mucin-4 (MUC4), claudin-4 are markers suggestive for epithelial metastasis [6]. Histologically, 70–85% of mesotheliomas are epithelioid, 10–25% are biphasic, 10% are sarcomatoid, and <2% are desmoplastic. More recently, a multidisciplinary group endorsed by IASLC (International Association for the Study of Lung Cancer)/EURACAN (European Reference Network on Rare Adult Cancers) reviewed the histologic classification of MPM considering recent molecular, immunologic, and therapeutic advances [7]. MPM has an ominous prognosis, with a life expectancy of 8–14 months in the population treated with chemotherapeutic agents and with a five-year survival rate that rarely encompasses 5% [8]. Recently, the introduction of combination immunotherapy, combined blockade of cytotoxic T-lymphocyte-associated antigen 4 (CTLA4) and programmed death 1 (PD1), in a naïve population impacted survival probability with median overall survival of 18.1 months and with three years overall survival rate of 23% compared to 15%, which was provided by traditional chemotherapy [9]. Even if this therapeutic strategy is practice-changing and opened a new avenue in the treatment of MPM, only a small number of patients can be treated effectively, mainly because the diagnosis is often delayed or missed. Despite clinical MPM diagnosis is easily suspected because the patients’ medical history and CT scan images are eloquent, sometimes it may be difficult and challenging, requiring a multidisciplinary approach through correlation of cyto-histologic aspects and clinical-radiological findings [10], since criticalities can potentially occur on every step of the diagnostic plan, from the clinical presentation to the pathologic findings. 

MPM is an incredibly interesting disease, and the continuous breakthroughs in its anatomical, pathologic and biomolecular aspects are going to allow a correct classification and open up modern therapeutic possibilities. Recently, Rossi et al. [11] pointed out the peculiarities of this disease and reviewed its wide spectrum of atypical clinical, radiological and morphological presentation. They reported singular clinical situations of MPM like those arising without apparent asbestos exposure, patients aged less than 40 years, and occurrence of paraneoplastic syndromes as onset scenarios. They presented a broad spectrum of radiological appearance: from typical diffuse pleural thickening and unilateral effusion to atypical pulmonary or mediastinal localized mass, rather than acute pleural empyema, chylothorax, or interstitial lung disease. Finally, they focused on unusual histological features of MPM with aberrant expression of mesothelial or epithelial markers. They concluded that MPM could occur with atypical presentations on every examined level, even in various combinations. We fully agree with that statement, and we think that ultimately this is exactly what makes MPM so interesting: a relative acknowledged disease with well-known and dramatic natural history, which can occur with unusual features, challenging different involved medical specialties in several new diagnoses. Therefore, we decided to share our own experiences on unusual MPM with the aim of increasing knowledge and focusing attention on this topic.

At our center, on average, 26 cases of MPM per year are diagnosed. Here, we present three cases with the unconventional and complex diagnostic process of MPM that we have experienced during routine practice in the last years. The first one is the glaring exemplification of the difficult differentiation between MPM and other pleural diseases. The second one is the example of an atypical and unexpected presentation of MPM, whilst the third one is a rare case of an MPM in situ.

## 2. Case Presentation and Discussion

### 2.1. Case 1

**Figure 1:**
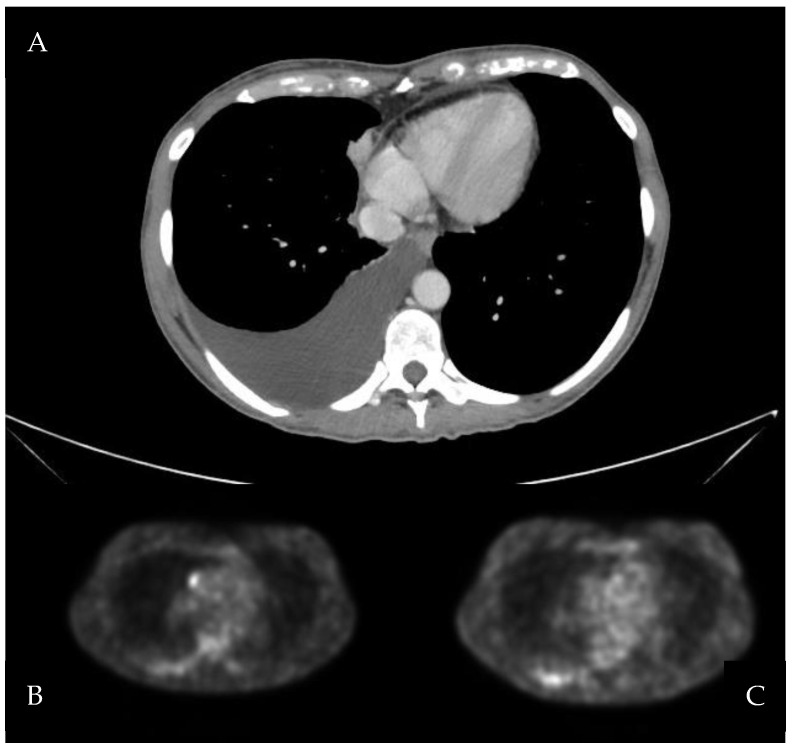
(**A**): axial section of CT scan showing right pleural effusion and ipsilateral nodular irregularities of parietal and mediastinal pleura. (**B**,**C**): axial sections of ^18^F-FDG-PET scan showing increased metabolic activity of right pleural nodules.

**Figure 2:**
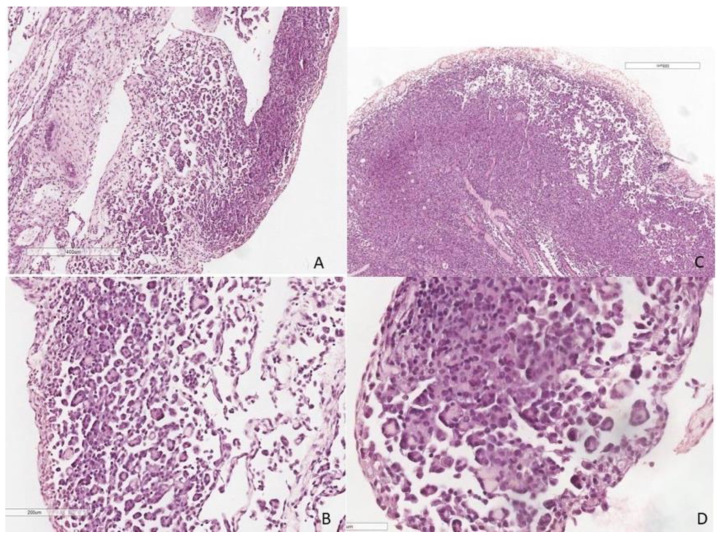
Histologic sections from multiple pleural biopsies; in (**A**,**B**,**D**) show evidence of mild cytologic atypia with an appearance of architectural complexity with micropapillary formation. In (**C**) note, admixture of mesothelial cells with histiocytes as “nodular histiocytes/mesothelial hyperplasia” (H&E, scaling as reported in images).

A 49-year-old woman presented to the Emergency Department (ED) for fatigue and abdominal pain. She was a non-smoker with a history of uterine fibromatosis, and seven months before, she had undergone laparotomic hysterectomy and salpingectomy for metrorrhagia, with histological examination resulting in leiomyomatosis with simple glandular hyperplasia without atypia. An abdominal ultrasonography was performed: right pleural effusion and ascites were found together with a right ovarian cyst and right hydronephrosis. A thorax-abdomen CT scan was consequently performed, confirming the sonography findings and revealing suspicious pleural nodules, as shown in Figure 1. The patient underwent urgent surgical right nephrostomy, and right pleural drainage was positioned. The analysis of the pleural effusion sample revealed malignant tumoral cells with signet-ring-like morphology. An ^18^F-fluorodeoxyglucose (FDG)-positron emission tomography (PET) scan was performed, resulting in tracer uptake in the right ovary and in pleural nodules. The FDG-absorbing ovarian cyst was consistent with a corpus luteum; esophagogastroduodenoscopy and colonoscopy were negative. A revision of the cytologic sample was performed in a tertiary center: diffuse-type gastric cancer with prevalent signet-ring cells was confirmed. The patient was therefore referred to our Thoracic Department (TD) for the management of pleural effusion. We performed a right thoracoscopy with random pleural biopsies, surprisingly without any macroscopic evidence of malignancy. Histological analysis of pleural biopsies, as presented in Figure 2, concluded that atypical pleural proliferation (WT1+, calretinin+, BAP1+, CK20-, PAX8-, CD31-) in a complex architecture, despite the absence of clear stromal or adipose tissues invasion, suggestive for epithelioid MPM. Thus, the sample was further analyzed through fluorescence in situ hybridization (FISH): p16 (9p21) was found to be homozygously deleted in 8% of the sample and heterozygous deleted in 67%. Then, the patient underwent a bilateral ovariectomy with a cytologic sample from peritoneal fluid and reimplant of the right ureter. The histologic result was negative for malignancy. Two weeks later, further thorax/abdomen CT was performed: the resolution of pleural effusion, no pleural nodules nor lung lesions, no pathological nodes in the abdomen, and minimal pelvic effusion were detected. Since the case was troubling and not conclusive for cancer, a pathologist’s second opinion (JR) was requested, who revised all the pleural histologic samples, concluding that atypical mesothelial and histiocytic proliferation consistent with the reactive process. At the two-year radiological follow-up, the patient was still alive without evidence of macroscopic disease.

The first case summarizes the extremely tricky differential diagnosis between MPM and other pleural affections. At first sight, all the clues pointed at a metastatic gastro-enteric or gynecological cancer, but further analysis lowered the chance of this entity, bringing into play MPM. Distinguishing metastatic cancer from primitive MPM relies on immunohistochemical investigations. Calretinin, WT-1, CK5, antibody against podoplanin (D2-40), mesothelin, and HEG1 are the most reliable markers of mesothelial differentiation [12]. On the other hand, transcriptional thyroid factor 1 (TTF-1) and napsine are selective for lung adenocarcinoma, CD10 and paired-box gene 8 (PAX8) for renal cell carcinoma, prostate specific antigen (PSA) for prostatic cancer, hormonal receptors for breast cancer, caudal type homeobox 2 (CDX2) and cytokeratin-20 (CK20) for gastrointestinal cancer, and p63 and p40 for squamous cancer [13]. Claudin-4 has been recently proven to be highly selective for epithelial neoplasms [14]. International Societies recommend employing two positive and two negative markers for differential diagnosis [15]. However, to further underline the difficulties, if still needed, in literature are reported cases of lung cancers expressing mesothelial markers, such as calretinin, mesothelin, or D2-40 [16]; on the other hand, there are experiences of CK20, TTF1, p40 or p63 expression in MPM [17]. After having hardly excluded a pleural metastatic cancer, the greatest criticism lies in differentiating MPM from reactive mesothelial hyperplasia. Indeed, mesothelial cells may appear really atypical in the case of a reactive inflammatory process, e.g., even after a chest tube is placed. Detection of pleural or lung parenchymal soft tissue invasion is a conclusive sign of MPM, but this may not always be evident. In these cases, some immunohistochemical markers may be helpful in supporting the differential diagnosis. A strong and circumferential pattern of Epithelial Membrane Antigen (EMA) expression is suggestive of MPM, and so it is p16 deletion [18]. Expression of Glucose Transporter 1 (GLUT-1) and Insulin-like growth factor II m-RNA-binding Protein 3 (IMP3) may be associated with MPM [19]. The combined use of these markers increases diagnostic reliability, but none of them is conclusive for MPM rather than mesothelial hyperplasia. Recently, the BRCA-1 associated protein 1 (BAP1) has been demonstrated to be the most reliable marker for MPM when unexpressed [20]. BAP1 is a gene that encodes ubiquitin C-terminal hydrolase engaged in keeping the proper ubiquitination of histones, recently demonstrated to act as a tumor suppressor gene modulating carcinogenesis [21]. In vitro studies proved that BAP1 loss induced cellular proliferation through up-regulation of Enhancer of Zeste Homolog 2 (EZH2) [22], although the ultimate role of BAP1 loss in mesothelial malignant transformation is still unclear. BAP1 status can be determined through IHC, and its loss has been detected in about 60% of MPM, mostly in the epithelioid subtype [23]. A meta-analysis involving 1824 cases of mesothelioma proved that nuclear BAP1 loss is fully specified for MPM versus reactive mesothelial hyperplasia [24]. Another promising marker is Methylthioadenosine Phosphorylase (MTAP), since the loss of its expression, which is a surrogate for CDKN2A (p16) deletion, showed high accuracy in confirming the diagnosis of MPM [25]. In our case, BAP1 was positive, whereas p16 was deleted in a significant part of the cellular population. In such situations, differentiating MPM from atypical mesothelial proliferation may be difficult, and often only a close clinical follow-up could distinguish between different pathological entities. The above-described case was really challenging and troubled our multidisciplinary team. The most likely option is that the ovarian cyst induced compression of the ureter leading to hydronephrosis, which caused ascites that, in turn, spilled over the diaphragm into the chest. The pleural nodules are hard to be explained, but they could represent the exemplification of how strong the reactive process of mesothelial cells can be.

### 2.2. Case 2

**Figure 3:**
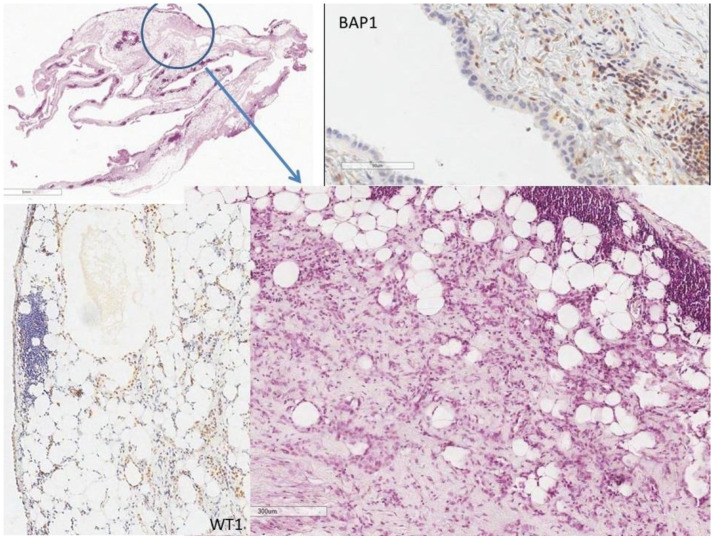
Low magnification from large pleural biopsy in case 2: infiltrative growth into subpleural adipose tissue is circled in blue, with evidence of WT1 positive structures surrounding fat cells and BAP1 defective in the same mesothelial cells. (H&E, scaling as reported in images).

A 77-year-old woman was admitted to our ED for dyspnea and right hemithorax stabbing pain. A chest X-ray found the right hydropneumothorax. She was referred to our TD. She was a non-smoker, without known exposure to asbestos, affected by hypertension, with a history of total thyroidectomy for goiter in 2014, complicated with postoperative myocardial infarction. Right pleural drainage was positioned with the evacuation of air and 2000 mL of serous fluid, whilst without complete resolution of pneumothorax. Thorax CT was performed, confirming residual right pneumothorax and identifying focal ground-glass opacities (GGOs) in the right upper lobe of probable inflammatory significance and sub-centimetric short-axis mediastinal nodes. She underwent right thoracoscopy: an apical dystrophic area of the lung was found, and therefore pulmonary apicectomy was performed. Pleurodesis was gained through partial pleurectomy and talc poudrage. Even if macroscopically, the pleura seemed to be affected by the inflammatory process without nodular involvement, histologic examination of the pleural samples revealed an atypical epithelioid mesothelial proliferation, well-differentiated, with prevalent tubular growth, focally extended to subpleural adipose tissue, consistent with epithelioid MPM (calretinin+, WT1+, BAP1-), as shown in Figure 3. After a multidisciplinary consult, three months after surgery, the patient received three courses of carboplatin + pemetrexed, followed by two courses with pemetrexed alone due to grade 2 anemia. Eight months after surgery, a follow-up CT scan showed locally progressive disease: basal and para-mediastinal right pleural thickening with contrast-enhancement, without signs of abdominal or peritoneal involvement. One year after surgery, the right pleural effusion relapsed. The patient underwent right pleural port-a-cath placement, and due to worsening clinical conditions, no second-line treatment was offered. She passed away sixteen months after the diagnosis of MPM.

The second case reports an unusual presentation of MPM. As usual, symptoms referred by patients are non-specific, including cough, dyspnea, and dull chest pain, due to pleural effusion and hemithorax shrink. People are usually investigated because of pleural effusion occurrence. In our case, the onset was characterized by a spontaneous unilateral hydropneumothorax, refractory to conservative management through chest drain placement with incomplete lung re-expansion. Primary pneumothoraxes are common, and the development of hydropneumothorax is not uncommon in the case of persistent pneumothorax. Pneumothoraxes are associated with malignancies in less than 0.02% of cases [26], and mesotheliomas can be associated with pneumothorax in up to 11% of cases [27]. Therefore, careful investigations are mandatory in case of failure of lung re-expansion after proper treatment. Based on the experience of Sheard et al. [27], among 91 patients with persistent spontaneous pneumothorax undergoing pleurectomy, 5 (4.3%) were diagnosed with MPM. According to Bright–Thomas and colleagues [28], the underlying mechanism at the base of pneumothorax in MPM is still unclear. Still, it may be related to irritation of the visceral pleural and invasion of the lung parenchyma or rupture of tumoral nodules. Another mechanism could be the presence of peripheral tumoral nodules, which lead to a ball-valve action overinflating the lung and forming fragile subpleural bullae [29]. The first pneumothorax complicating MPM was reported by Eisenstadt in 1956 [30]. In 2000, Alkhuja et al. [31] reported the first case series of four patients with MPM presenting as spontaneous pneumothorax. As stated by Prasad and colleagues in 2013 [32], until then, in the literature, less than 35 cases of MPM presenting as spontaneous pneumothorax have been collected. Mitsui et al. [33], reviewing Japanese literature, reported a total of 16 MPM patients with spontaneous pneumothorax: 81% were male, and concurrent pleural effusion was present in 56% of cases. Recently, Sattar and colleagues [34] focused exclusively on patients with MPM presenting as hydropneumothorax. In their literature review, nine cases were collected: a right-sided predominance was observed, exclusively related to the male population, mainly with significant tobacco history. The median survival was 16 months, consistent with that recorded in our case. Albeit it is henceforth known that pneumothorax could occur throughout the course of the disease, it is still hard to promptly diagnose mesothelioma when presenting as pneumothorax or hydropneumothorax. Therefore, MPM should always be retained as a possible diagnosis, and it is worth acting accordingly since an early-stage diagnosis could allow for multimodality treatment, ensuring the best prognosis. Unlikely, as our case shows, the natural history of MPM could be really nefarious even when promptly recognized and treated.

### 2.3. Case 3

**Figure 4:**
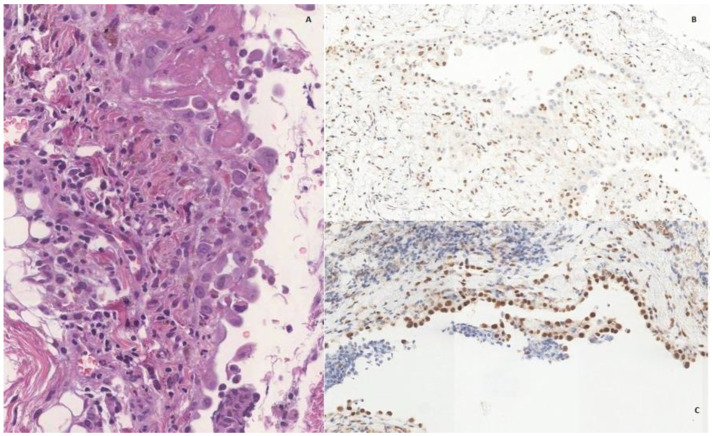
In (**A**), mesothelial lining cells with mild to moderate cytologic atypia, without infiltration of tissues below; no evidence of infiltrating component even with WT1 IHC (**C**), while BAP1 is defective (**B**), some non-neoplastic mesothelial reactive cells are intermixed.

A 70-year-old man with a history of dilated cardiomyopathy and coronary heart disease presented to the ED for thoracic pain. He was a farmer without known exposure to asbestos. Acute cardiac disorders were promptly ruled out. A chest X-ray revealed a right pneumothorax. A right pleural drain was positioned. Due to prolonged air leaks, he later underwent right pulmonary apicectomy, partial pleurectomy, and talc poudrage through right thoracoscopy, without evidence of macroscopic pleural disorders. Histologic examination of pleural sections showed a single layer of epithelioid mesothelial cells (WT1+, calretinin+) with mild atypia and defective expression of BAP1, without evidence of adipose or lung tissues invasion, as presented in Figure 4. Since the absence of radiologic or direct signs of neoplasia, according to the latest WHO guidelines [35], a diagnosis of MPM in situ was made. Chest X-ray performed at one month revealed residual right hydro-pneumothorax. The patient underwent right thoracic drain positioning and talc slurry. He was discharged with a Heimlich valve, and the thoracic drain was removed after ten days as soon as the air leaks ceased. At the six-month follow-up, he was asymptomatic and in good clinical conditions; at the one-year CT scan, he was still free from macroscopic pleural disease.

The third case is focused on the debated newest entity of MPM in situ. It was first conceptualized in 1992 as the replacement of pleural surface by neoplastic malignant mesothelial cells [36]. Originally, MPM in situ was intended more as a way to explain the pathogenesis of malignant mesothelioma rather than a clinical stage of diagnosis itself [37]. It was found in patients who presented the invasive form at once, thus possibly representing the spread of the latter through the pleural surface rather than its early phase. Moreover, reactive mesothelial cells might appear atypical, therefore making improbable the morphological differentiation between in situ neoplastic and reactive mesothelial proliferation. Topic-interested pathologists agreed on the impossibility of identifying malignant mesothelioma in situ on biopsies, hence contraindicating the diagnosis of MPM in situ. Modern developments in molecular pathology have allowed MPM in situ to be diagnosed in a multidisciplinary setting through a combination of clinical, imaging, morphological, and immunohistochemical data, as firstly reported by Churg and colleagues [38] applying the following criteria: (1) exclusively surface proliferation of mesothelial cells with BAP1 loss on biopsy; (2) no evidence of tumor on imaging or by inspection of the pleura; (3) no invasive mesothelioma diagnosed for at least one year. The development rate of invasive MPM was 70% (7/10 patients) at an interval from 12 to 92 months (median 60 months) since the detection of MPM in situ. The crucial point underlined in that case series enrolling ten patients (nine with pleural mesothelioma, one with peritoneal mesothelioma) was that a surface proliferation of mesothelial cells with loss of BAP1 should be reported as MPM in situ [39]. The MPM in situ entity has gained more and more consensus, enough to be included in the most recent WHO classification of Thoracic Tumors, as updated in 2021 [35]. The criteria introduced by Churg and colleagues have been recently adapted and also recommended by the 2021 ESMO Clinical Practice Guidelines [40], even though without providing insights about the management of this entity. We believe that the concept of MPM in situ is still somewhat controversial since its diagnosis is finally based exclusively on BAP-1 loss, whose reliability is not full. Furthermore, a one-year follow-up could be too short to correctly differentiate between the very early presentation of MPM or reactive pleuritis. Until further experiences are collected, and clear recommendations are given, in case of doubt, we suggest pursuing a conservative strategy and strictly following up with the patient. 

## 3. Conclusions

MPM is a fascinating disease with known pathogenetic factors and an ignominious prognosis. Its diagnosis always requires a multidisciplinary team, and even if its classic features are now well-known, atypical clinical, radiological, or morphologic presentations are increasingly becoming the rule, challenging healthcare workers throughout the world. The following key messages can be outlined from our experience: (1) despite great advances made in recent years, definitive biomarkers to confidently distinguish MPM from other pleural affections are still sought; (2) patients presenting with persistent unilateral hydropneumothorax must always be further investigated for MPM, even if without a known history of asbestos exposure or smoking habit; (3) MPM in situ is now a reality, and long-term follow-up and management of this new entity are required to understand if natural history of the disease can be revolutionized by intervening in its early phase.

## Data Availability

The data presented in this study are available on request.
